# Impact of static magnetic field exposure on *Stim1* and *Itpr3* expression in hepatic cells of obese mice

**DOI:** 10.5455/javar.2025.l890

**Published:** 2025-03-25

**Authors:** Puji Sari, Rahma Nur Istiqomah, Luluk Yunaini

**Affiliations:** 1 Department of Medical Biology, Faculty of Medicine, Universitas Indonesia, Jakarta, Indonesia; 2 Master’s Programme in Biomedical Science, Faculty of Medicine, Universitas Indonesia, Jakarta, Indonesia

**Keywords:** Static magnetic fields, calcium, *Stim1*, *Itpr3*, obese mouse

## Abstract

**Objectives:**

This study investigates the effects of 2 mT static magnetic field (SMF) exposure for 1 h on the expression of *Stim1* and *Itpr3* genes in hepatic cells of obese mice. By examining these critical regulators of calcium (Ca^2+^) signaling and cellular metabolism, the research aims to elucidate the role of SMF in modulating molecular pathways essential for Ca^2+^ homeostasis and metabolic regulation in the context of obesity.

**Materials and Methods:**

Liver samples were obtained from C57BL/6J mice and preserved in RNALater. The samples were divided into two main groups: the control group, which received a standard diet, and the obese group, which was exposed to a high-fat diet. Furthermore, the obese group was stratified based on the duration of SMF exposure, including intervals of 0, 2, 7, 14, and 21 days (1 h per day with an intensity of Bmax = 2 mT). Statistical tests were conducted with a significance level of *p *< 0.05.

**Results:**

The research findings highlighted a noteworthy increase in the relative expression of *Stim1* and *Itpr3* among obese mice exposed to SMF for 7 days (obe7) and those exposed for 14 days (obe14) in comparison to the obese group without SMF exposure. Both the obe7 and obe14 groups exhibited no significant difference in relative *Stim1* expression when compared to the non-obese group. However, in terms of *Itpr3 *expression, the obe14 group did not show a significant difference from the non-obese mouse group. The results of the correlation analysis unveiled a substantial and robust correlation between the relative expression of Stim1 and Itpm3 (*r *= 0.627, *p *< 0.001).

**Conclusion:**

These findings suggest a potential link between SMF exposure, the expression of Ca^2+^ regulatory genes, and the intricate pathways involved in obesity-related molecular responses.

## Introduction

Calcium (Ca²⁺) functions as a widespread intracellular messenger, overseeing a myriad of cellular activities; however, excessive levels can lead to toxicity and cell demise [1]. The endoplasmic reticulum (ER) and, in muscle cells, the sarcoplasmic reticulum serve as the principal internal reservoirs for Ca²⁺ [2,3]. The pivotal role of Ca²⁺ signaling extends to fundamental cellular processes encompassing transepithelial transport, differentiation, oocyte fertilization, gene transcription, muscle contraction, learning and memory, metabolism, secretion, motility, and membrane stimulation [4]. Moreover, the modulation of intracellular Ca^2+^ levels plays a crucial role in both cell formation and apoptosis, orchestrating essential biological functions like gene expression, cell proliferation, and cell death [5]. The intracellular Ca^2+^ concentrations can dynamically vary, either decreasing or increasing based on physiological demands. As a divalent cation, Ca^2+^ can be acquired through various proteins, receptors, and ion channels [6]. Ca²⁺ signals are derived from ion channels, encompassing both plasma membrane and intracellular channels, and are regulated by diverse Ca²⁺ pumps, transporters, and buffers. The intricate Ca^2+^ signaling pathway involves multiple components, and any dysfunction in this pathway is implicated in the pathogenesis of various diseases [7].

Ca²⁺ storage within cells primarily resides in the ER. The release of Ca²⁺ is facilitated by ryanodine receptors (RyRs) and inositol 1, 4, and 5-triphosphate (InsP3) receptors (ITPRs) [8]. ITPR encompasses three isoforms, namely ITPR1, ITPR2, and *Itpr3*, each encoded by distinct genes. Despite being encoded by different genes, these isoforms share a 70% homology in their primary sequence. These isoforms can form functional channels by assembling into either homo- or heterotetramers [9]. Specifically, *Itpr3* responds to varying Ca²⁺ concentrations, with lower concentrations stimulating channel conductance and higher concentrations inhibiting channel conductance [10,11].

The ER serves as a source of Ca^2+^ signals that regulate the process of Ca^2+^ entry into cells, thereby maintaining cellular homeostasis in eukaryotic cells through store-operated calcium entry (SOCE). SOCE is governed by the ER membrane protein Stromal Interaction Molecule 1 (*Stim1*) following a reduction in cellular Ca^2+^. *Stim1* acts as a Ca^2+^ sensor and regulatory protein localized in the ER [12–15]. Upon receiving a signal from *Stim1*, the inositol 1,4,5-trisphosphate receptor (*Itpr3*) facilitates the influx of Ca²⁺. This receptor manages the entry and exit of Ca²⁺ to uphold cellular Ca^2+^ homeostasis [16]. According to Caro's research in 2020, SHYS5 cells experiencing a *Stim1* knockout condition exhibited a decrease in *Itpr3* expression. This gene receives signals for Ca²⁺ release from the ER to the cytosol [17]. Activation of ITR3 by IP3 causes Ca²⁺ efflux from the ER lumen to the cytoplasm. This decrease in Ca²⁺ in the RE lumen is sensed by the interaction of stromal Molecule 1 (*Stim1*), a transmembrane receptor in the ER. Low Ca²⁺ concentrations in the ER lumen trigger a conformational change in *Stim1* that causes it to associate with the plasma membrane-bound channel protein ORAI1, forming a calcium release-activated channel (CRAC) and causing Ca²⁺ influx into the cytoplasm [18]. While the increase in Ca^2+^ is influenced by binding proteins, various cell stimuli, such as membrane depolarization, extracellular signaling molecules, or intracellular messengers, can induce an elevation in Ca²⁺ levels [19].

Aberrations in Ca^2+^ signaling pathways are implicated in various metabolic and cellular dysfunctions, including those associated with obesity [20]. In hepatic cells, these pathways are crucial for metabolic regulation and cellular function [21]. Emerging evidence suggests that external stressors, such as Static Magnetic Fields (SMFs), can influence Ca^2+^ dynamics. SMF, widely utilized in biological research, has been shown to modulate voltage-gated ion channels and intracellular Ca^2+^ levels [22,23]. However, the specific molecular mechanisms underlying these effects remain poorly understood.

Recent findings indicate that SMF exposure at 2mT intensity for 1 h increases Ca^2+^ levels in the livers of obese mice [23,24]. Despite this, the precise impact of SMF on key regulatory genes in Ca^2+^ signaling, such as *Stim1* and *Itpr3*, has not been explored. This knowledge gap is critical, as *Stim1* and *Itpr3 *are central to Ca^2+^ homeostasis and metabolic regulation. Their expression levels may provide insights into how SMF influences cellular pathways, potentially uncovering therapeutic opportunities for addressing metabolic disorders in obesity.

In this study, we aim to fill this gap by investigating whether 2mT SMF exposure affects the expression of *Stim1* and *Itpr3* in the hepatic cells of obese mice. By focusing on these key genes, this research provides a novel perspective on the interplay between external magnetic fields and intracellular Ca^2+^ regulation, offering potential implications for metabolic dysfunction therapy in obesity.

## Materials and Methods

### Ethical approval

This research has undergone ethical review by the Health Research Ethics Committee, Faculty of Medicine, Universitas Indonesia, under the registration number KET-868/UN2.F1/ETIK/PPM.00.02/2023.

### Time and place

This study was categorized as experimental research, designed to investigate the expression of *Stim1* and *Itpr3* in the liver of mice. The research spanned from July 2023 to September 2023 and took place at the iRATco Animal Laboratory, Medical Biology Laboratory, and IMERI FKUI.

### Research sample

The samples in this study comprised male C57BL/6J mice weighing 20 gm and 6 weeks old, sourced from the iRATco Animal Laboratory. Subsequently, the mice underwent acclimatization for 5–7 days at a temperature of 23°C. The mice were then categorized into six groups. One group served as non-obese (control) and was fed a standard diet, while the remaining five groups, designated as the obese groups, were fed a high-fat diet. The obesity group was further subdivided based on the duration of exposure, labeled as exposure smf mice obesity 0 day (Obe0) (without exposure SMF), Obe2 (exposure SMF 1 h/day for 2 days), Obe7 (exposure SMF 1 h/day for 7 days), Obe14 (exposure SMF 1 h/day for 14 days), and Obe21 (exposure SMF 1 h/day for 21 days). The allocation of mice in each group followed the Federer formula for group size determination.

(t−1)(n−1) ≥ 15

(6−1)(n−1) ≥ 15

5n−5 ≥ 15n ≥ 4

Note: t = Number of treatments,

 n = sample size per group.

The obese group of mice was subjected to a high-fat diet (HFD) for approximately 10 weeks until they developed obesity. Throughout this period, food consumption and body weight of the mice were closely monitored every week. Obesity was evaluated using criteria such as weight gain or the Lee obesity index, calculated by the formula body weight (g^0.33) ÷ naso-anal length (mm). Mice were classified as obese if their index value exceeded 310 [25]. The SMF exposure procedure for the obese group involved daily 1h sessions with an intensity of Bmax = 2 mT. Following exposure, depending on their respective groups, the mice were euthanized, and their livers were isolated and preserved in an RNALater solution.

### RNA isolation

Isolation was conducted utilizing the spin column method with the Zymo Research kit. A liver sample, previously preserved in RNALater, weighing 0.3 gm, was taken and placed in a microtube. Subsequently, 300 µl of DNA Shield was added to the sample. Homogenization was then performed using a homogenizer. The subsequent steps followed the KIT instructions. The results of RNA isolation were validated using a nanodrop and stored in a freezer at −80°C.

### Gene expression analysis using qRT-PCR

Gene expression analysis using qRT-PCR in this study was performed utilizing an Applied Biosystems® 7500 Fast machine and materials from the SensiFASTTM SYBR® Lo-Rox Two-Step Kit. The isolated mRNA underwent reverse transcription into cDNA using the ReverTra Ace qPCR RT Master Mix kit. Primers for *Stim1 *were designed using the NCBI Primer designing tool software (Tabel 1). This mixture was then subjected to two repetitions (Duplo) in the PCR machine. *Stim1* and *Itpr3 *expression were calculated using the Livak formula, with the β-actin gene serving as the housekeeping gene. After confirming the purity of the RNA, cDNA was prepared to proceed to the RT-PCR process. The expression results were analyzed using the Livak formula.

ΔCT sample (Obes0, −2, −7, −14, −21) = CT target gene–CT reference gene

ΔCT calibrator (normal) = CT target gene–CT *r* reference gene

ΔΔCT = ΔCT–ΔCT calibrator

Expression relative gen = 2–ΔΔCT

### Statistical analysis

Statistical analysis was conducted using SPSS 27 for Windows. Each gene expression variable underwent tests for normality and homogeneity. A normality test was applied to each group, with the criteria for normally distributed and homogeneous data set at *p *> 0.05. If the obtained data exhibited both normal distribution and homogeneity, subsequent analyses included the paired t-test to assess the impact on reducing the Lee index, the ANOVA test to examine gene expression, and the correlation test, conducted using the Spearman test. The significance level was determined with a value of *p *< 0.05.

## Results

The effect of SMF led to a reduction in the Lee index value within the obese group (Table 2). The data for the Lee index in mice, both before and after exposure, exhibited a normal distribution. Consequently, the statistical analysis employed the parametric paired *T*-test. According to the paired T-test, the Lee index values before and after exposure exhibited significance (*p *< 0.05) in the control, Ob0, and Ob14 groups. Both the normal and Ob0 groups displayed a significant increase compared to the initial values. Conversely, the exposure group demonstrated a tendency toward a decrease in the Lee index, with only the Ob14 group experiencing a significant reduction, although the decrease was not significant in other groups.

**Figure 1.**
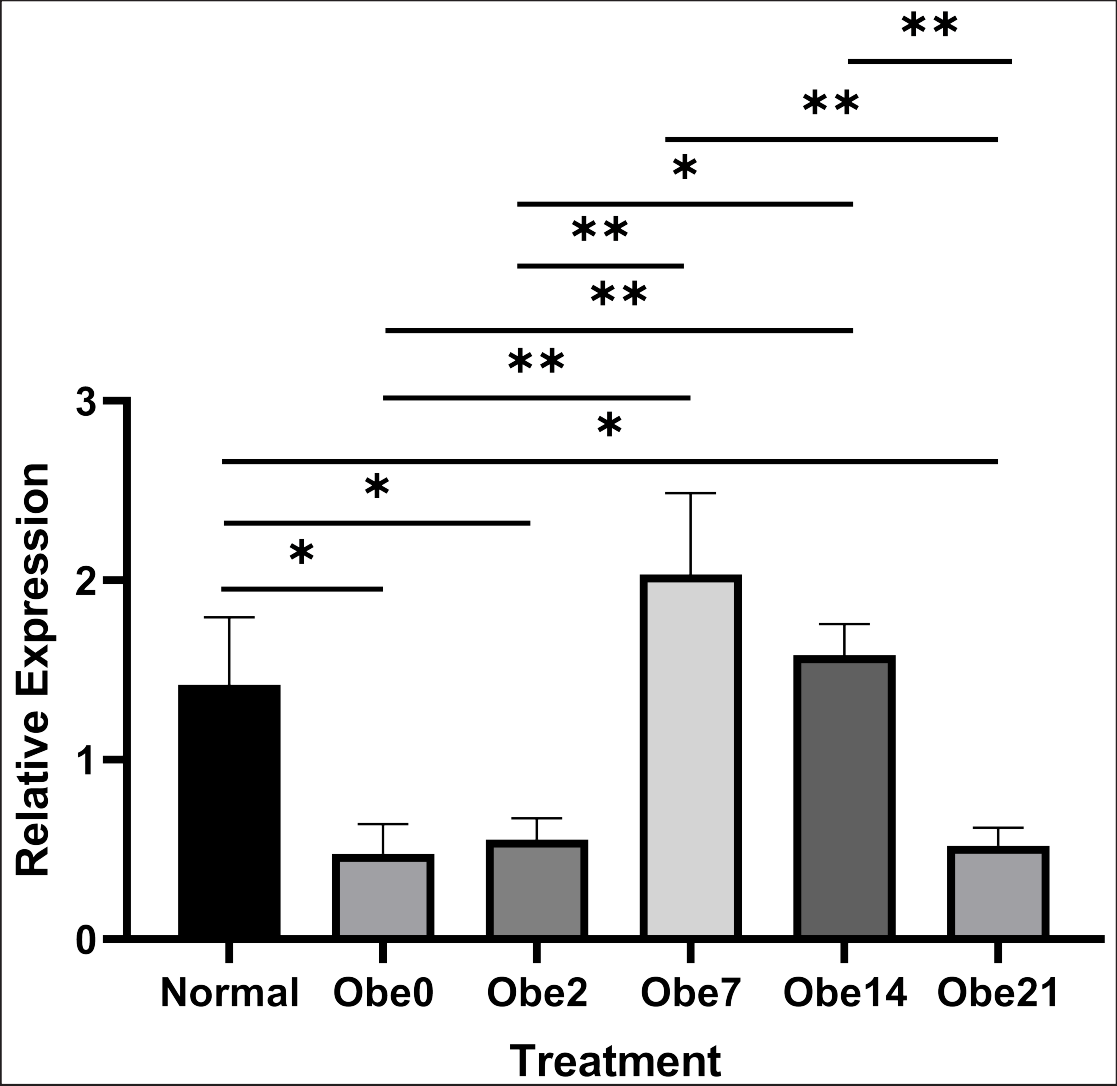
The elevation in the relative expression of *Stim1* in obese mice subjected to SMF was statistically analyzed through a one-way ANOVA test (*p* < 0.001), followed by post hoc testing. The significance levels are denoted as *p < 0.05 and **p < 0.01. Normal: non-obese mice; Obe: obese mice; 0,2,7,14,21: duration of SMF exposure in the day.

**Figure 2.**
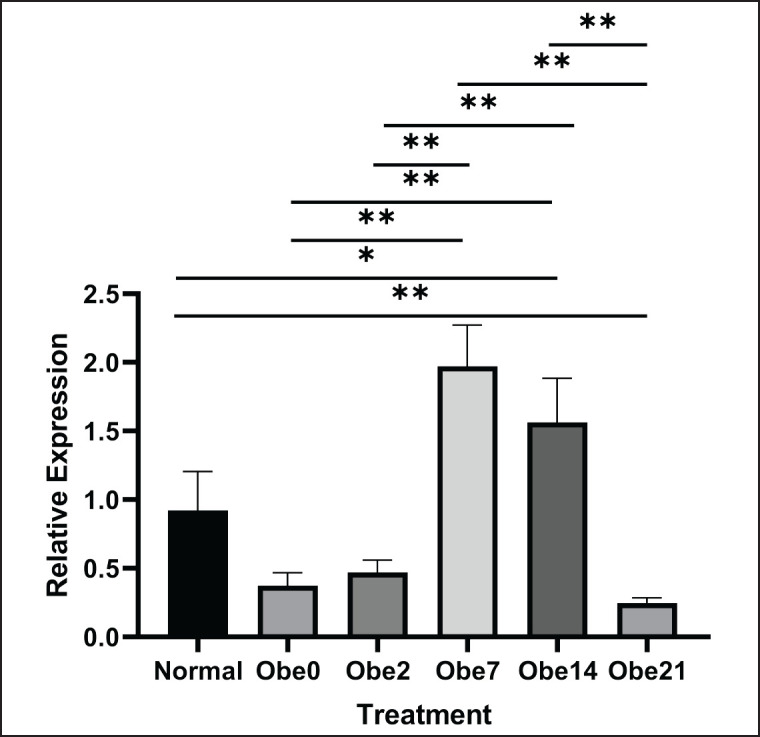
Relative expression of *Itpr3* expression after SMF exposure in obese mice. The statistically analyzed using a one-way ANOVA test (*p* < 0.001), followed by post hoc testing. The significance levels are denoted as *p < 0.05 and **p < 0.01. Normal: non-obese mice; Obe: obese mice; 0,2,7,14,21: duration of SMF exposure in the day.

The outcomes of the relative gene expression analysis for *Stim1 *and *Itpr3*, computed using the Livak formula, were illustrated in Figure 1 for *Stim1* and Figure 2 for *Itpr3 *expression. Statistical analysis of *Stim1* expression utilizing the ANOVA test yielded a result of 0.001, which is less than *p *< 0.05. This outcome indicated significant variations in gene expression among the groups. Subsequently, a Post Hoc test was conducted, revealing notable differences between non-obese and Ob0, Ob2, and Ob21 groups. Meanwhile, within the exposure group, a significant distinction was observed between the Ob0 group and Ob7 and Ob14, with a significance value of <0.05. The Ob0 group, obese without exposure, exhibited the lowest expression compared to the others, suggesting that SMF exposure might enhance *Stim1* expression. The Ob7 group displayed the highest relative gene expression, with a tendency to decrease in Ob14. A similar pattern was observed in the results of relative gene expression for *Itpr3*.

**Table 1.** Primer sequences for analysis relative expression using qRT-PCR technique.

Gene	Sequences
*Stim1*	F: 5'-AAGACCTCAATTACCATGACCC-3' R: 5'-AGTTGTACACTTCTGATGACTTCC-3'
*Itpr3*	F: 5'-TGCCTTTGATTCCTCCACTG-3' R: 5'-CTGCTGGTCTTCCCTACTCC-3'
*Beta-actin*	F: 5'-CTCCCTGGAGAAGAGCTATGA-3' R: 5'-CCAAGAAGGAAGGCTGGAAA-3'

Note: F: forward, R: reverse

**Table 2.** Pre and post-Index Lee after exposure SMF.

Group	Index Lee		Nilai p
Mean ± SD	
Pre	Post
Non-obese	256 ± 0.146	271 ± 0.220	0.038*
Ob0	339 ± 0.009	353 ± 0.180	0.045*
Ob2	340 ± 0.337	338 ± 0.348	0.254
Ob7	345 ± 0.20 1	344 ± 0.021	0.081
Ob14	342 ± 0.021	340 ± 0.022	0.038*
Ob21	373 ± 0.051	366 ± 0.054	0.053

Note: * significant difference between pre and post using *T*-Test dependent.

The findings indicated that *Itpr3* expression of the Ob7 group exhibited values that tended to be higher compared to controls and other obese groups, as depicted in Figure 2. The results illustrate that following an increase in *Itpr3* expression in the Ob7 group, there was a subsequent tendency for a decrease in Ob14. Based on the ANOVA results, the obtained value was <0.05, indicating significant differences between the groups. The relative expression of the *Itpr3* tended to be higher in the non-obese group compared to the obese without SMF exposure group.

**Figure 3.**
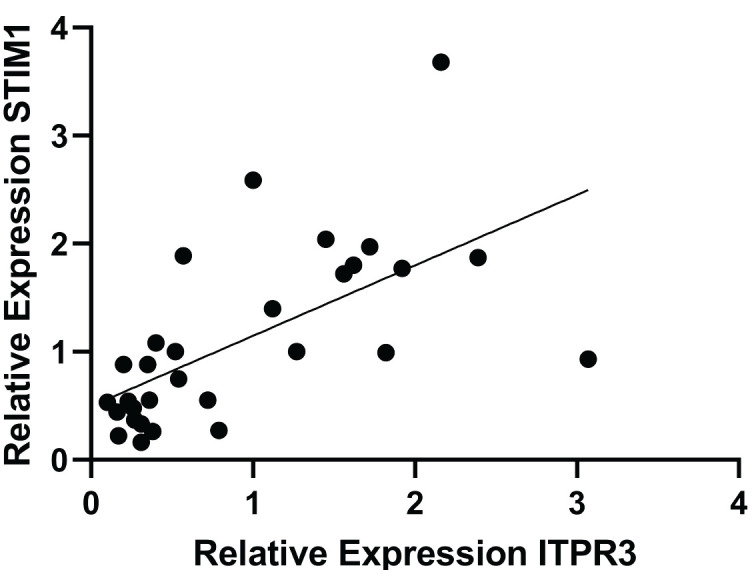
A scatter plot was created to illustrate the correlation between the *Itpr3*(variable X) and the *Smit1* (variable Y). The data was analyzed using the Pearson correlation test, yielding a significant result with *p *< 0.001 and a correlation value of *r *= 0.627.

A correlation test analysis between the relative expression of *Stim1* and *Itpr3* was conducted using the Pearson test, yielding a correlation coefficient value of 0.627 with *p *< 0.05, indicating a strong and positive significant correlation. Therefore, if there is an increase in *Stim1* expression, it will be followed by an increase in *Itpr3 *expression, and conversely, if there is a decrease in *Stim1* expression, it will be followed by a decrease in *Itpr3 *expression. The data distribution between gene expressions is depicted in Figure 3.

## Discussion

Exposure to SMF can influence a reduction in Lee index values, which serves as an obesity indicator. Despite lacking statistical significance, each SMF-exposed group exhibited a decrease in the Lee index value. This finding aligns with the research conducted by Sari et al. [26], where obese mice subjected to SMF displayed weight loss. The weight reduction was attributed to higher adipose tissue density in the normal group compared to SMF-exposed mice, leading to a decrease in density—an avenue that can contribute to weight loss. Weight loss, in turn, has the potential to alter metabolic and inflammatory characteristics, facilitating the clearance and storage of ingested energy [27].

Exposure to SMF also impacts the expression of genes regulating intracellular Ca^2+^ levels in cells. The magnetic effect of SMF induces membrane depolarization, leading to the migration of more positive ions, including Ca^2+^, potassium, and sodium, compared to situations without SMF exposure [23]. The presence of Ca^2+^ ions can influence the efficacy of increasing gene expression. Istiqomah et al. [24] found an increase in intracellular Ca^2+^ ions in obese mice after 7 days of exposure with an intensity of 2mT for 1 h, followed by a decrease on the 14^th^ and 21^st^ days of exposure [24]. Elevated Ca^2+^ levels can significantly impact *Stim1* expression, leading to an increase in cytosolic Ca^2+^ concentration [28]. A similar effect is observed with *Itpr3*, where expression increases with rising Ca^2+^ levels [29]. This research aligns with the presented study, demonstrating an increase in the relative gene expression of *Stim1 *and *Itpr3 *after exposure to the obese group. However, the expression of *Stim1 *and *Itpr3 *is also influenced by the duration of exposure, with a peak in effectiveness at a specific time, namely Ob7. Compared to controls, Ob7 exhibited a 1.5x higher increase. The effect of exposure, when compared to the Ob0 group without exposure, resulted in a 1.25x increase in Ob2 and a 5x increase in Ob7 and Ob14, but a decrease in Ob21. While increased Ca^2+^ can have positive consequences by boosting gene expression, excessive elevation may lead to cell metastasis [30]. Therefore, this study confirms that high Ca^2+^ levels influence increased *Itpr3* expression, while conversely, low Ca^2+^ levels result in decreased *Itpr3* expression. This could be attributed to the possibility that excessive pressure induces exaggerated membrane depolarization, impacting intracellular Ca^2+^ levels [31]. By limiting chromatin accessibility to *Itpr3*, which encodes the Ca²⁺ channel, *Itpr3* deficiency results in reduced expression, resulting in impaired Ca²⁺ transfer from the ER to mitochondria required for induction of apoptosis [32].

These findings may be pertinent to this study because the Ob7 group, exposed for 7 days, exhibited an increase in *Stim1* and *Itpr3* expression, followed by a decrease in relative gene expression on days 14 and 21. The presence of Ca^2+^ may be modulated by the expression of *Stim1* and *Itpr3* genes. Neumann et al. [30] demonstrated that inherited *Itpr3* variants can modify Ca²⁺ signaling responses. Caro et al. [17] asserted a relationship between *Stim1* and *Itpr3* in influencing intracellular Ca^2+^ ion levels, stating that a reduction in *Stim1* gene expression impacts a decrease in *Itpr3* expression. These findings align with the current research, suggesting a formation of a similar pattern in the expression of *Stim1* and *Itpr3*, characterized by an increase in the Ob2 group, a peak in Ob7, and a decline in the Ob14 and Ob21 groups. Although statistical calculations do not establish a correlation, the data suggests a tendency toward a consistent pattern.

The observed pattern in *Stim1* and *Itpr3* expression implies that the effect of SMF exposure is contingent on the duration of exposure. The optimum duration, as determined in this study, for enhancing *Stim1* and *Itpr3* expression was 7 days, with an exposure duration of 1 h per day. Relative gene expression of *Stim1* and *Itpr3* in the obese group increases post-exposure, reaching its peak at Ob7, diminishing at Ob14, and further declining at Ob21. This indicates that the impact of SMF exposure is influenced by the exposure duration. Further research on the mechanism of Ca^2+^ reduction is crucial to comprehensively understand the effect of SMF exposure on mitigating degenerative diseases.

Despite providing valuable insights into the impact of SMF on obesity and Ca^2+^ gene expression, this study has some limitations. First, the sample size is relatively small, which may affect the generalizability of the findings. Additionally, the study focuses solely on the expression of *Stim1* and *Itpr3* genes, leaving other potential pathways and molecular targets unexplored. Furthermore, the lack of long-term follow-up beyond 21 days limits the understanding of the sustained effects of SMF exposure on gene expression and metabolic pathways. Future research should address these limitations by incorporating larger sample sizes, including additional molecular targets, and extending exposure durations to better evaluate the long-term implications of SMF exposure.

The correlation between relative gene expression between *Stim1* and *Itpr3* is very high, although more research needs to be done on the correlation of these two genes. However, the role of these two genes in intracellular Ca^2+^ intake in the ER is enormous. In research conducted by Zheng et al. [34], it was shown that a decrease in *Itpr3* gene expression also showed a decrease in Ca²⁺. A decrease in intracellular Ca²⁺ levels also supports the decrease in gene expression. Research conducted by Ge et al. [35] states that a decrease in Ca^2+^ affects *Stim1 *gene expression. Meanwhile, in experimental animal models given *Stim1 *overexpression, Ca^2+^ will increase, which is related to the activation of tumor cells. So, the correlation between the *Stim1* and *Itpr3* genes is related to the presence of Ca^2+^, which, if overexpressed, is likely to cause dangerous things.

## Conclusion

These results suggest that there may be a connection between being exposed to SMF, the activity of genes that control Ca^2+^ levels, and the complex networks that play a part in molecular responses related to obesity. Researching and understanding these connections further might help us figure out how the changes in gene expression happen and what they mean for obesity and metabolic processes that are connected.

## List of abbreviations

SMF, static magnetic field; Obe0, exporsure smf mice obesity 0 day; Obe2, exporsure smf mice obesity 2 day; Obe7, exporsure smf mice obesity 7 day; Obe14, exporsure smf mice obesity 14 day; Ca^2+^, calcium; ER, endoplasmic reticulum; RyRs, ryanodine receptors; InsP3, iinositol 1, 4, and 5-triphosphate; ITPR, inositol 1, 4, and 5-triphosphate; SOCE, store-operated calcium entry; STIM1, stromal interaction molecule 1; CRAC, calcium release-activated channel; EMF, electromagnetic fields; VGC, voltage-gated ion channels; ORAI1, gene encoded calcium release-activated calcium channel protein 1; HFD, high-fat diet; SPSS, statistical package for the social sciences.
